# The ESF Programme on Functional Genomics Workshop on ‘Data Integration in Functional Genomics: Application to Biological Pathways’

**DOI:** 10.1002/cfg.389

**Published:** 2004-03

**Authors:** Pierre-Alain Binz, Henning Hermjakob, Paul van der Vet

**Affiliations:** 1 Proteome Informatics Group Swiss Institute of Bioinformatics 1 Michel Servet Geneva 1211 Switzerland; 2 Department of Biological Sciences and Bioinformatics University of Geneva Geneva Switzerland; 3 European Bioinformatics Institute Hinxton Cambridge UK; 4 Department of Computer Science University of Twente Enschede The Netherlands

## Abstract

We report from the second ESF Programme on Functional Genomics workshop on Data Integration, which covered topics including the status of biological pathways databases in existing consortia; pathways as part of bioinformatics infrastructures; design, creation and formalization of biological pathways databases; generating and supporting pathway data and interoperability of databases with other external databases and standards. Key issues emerging from the discussions were the need for continued funding to cover maintenance and curation of databases, the importance of quality control of the data in these resources, and efforts to facilitate the exchange of data and to ensure the interoperability of databases.


**Comparative and Functional Genomics**
*Comp Funct Genom* 2004; **5**: 148–155.
Published online in Wiley InterScience (www.interscience.wiley.com). **DOI:** 10.1002/cfg.389

**Conference Editorial**

### The ESF Programme on Functional Genomics workshop on ‘Data Integration in Functional Genomics: Application to Biological Pathways’†

Pierre-Alain Binz[1,2]*, Henning Hermjakob[3] and Paul van der Vet[4]

[1] *Swiss Institute of Bioinformatics, Geneva, Switzerland*
[2] *Department of Biological Sciences and Bioinformatics, University of Geneva, Geneva, Switzerland*
[3] *European Bioinformatics Institute, Hinxton, Cambridge, UK*
[4] *Department of Computer Science, University of Twente, Enschede, The Netherlands*

**Correspondence to: Pierre-Alain Binz, Proteome Informatics Group, Swiss Institute of Bioinformatics, 1 Michel Servet, 1211 Geneva, Switzerland. E-mail: Pierre-Alain.Binz@isb-sib.ch*

*† Reproduced by permission of the European Science Foundation.*

### Abstract

**We report from the second ESF Programme on Functional Genomics workshop on Data Integration, which covered topics including the status of biological pathways databases in existing consortia; pathways as part of bioinformatics infrastructures; design, creation and formalization of biological pathways databases; generating and supporting pathway data and interoperability of databases with other external databases and standards. Key issues emerging from the discussions were the need for continued funding to cover maintenance and curation of databases, the importance of quality control of the data in these resources, and efforts to facilitate the exchange of data and to ensure the interoperability of databases. Copyright © 2004 John Wiley & Sons, Ltd.**

### Introduction

The integration of heterogeneous data and information is a key issue in the field of functional genomics. Currently available technologies are producing floods of results that have to be stored, interpreted, validated and correlated with biological significance. To this end, many databases have been created, some of which collect information on protein–protein interactions and biological pathways.

A first workshop on ‘Data Integration in Functional Genomics and Proteomics’ was held in Geneva in October 2001 [2,6], within the framework of the European Science Foundation (ESF) Programme on Integrated Approaches for Functional Genomics [4]. The goal was to bring together scientists with different backgrounds (biologists and bioinformaticians) who were participating in projects involving or requiring integration of heterogeneous biological data. The theme of ‘data integration requirements’ in the framework of general functional genomics approaches was extensively discussed, with a particular focus on proteomics-related questions; this rapidly evolving area is a good example of data heterogeneity.

One outcome of the general discussion was the proposal to organize another workshop that would focus on a more specific aspect of data integration issues.

The second Geneva workshop, which is reported here and in a number of separate contributions published in this special section, was therefore focused on the topic of data integration applied to the description, interpretation and understanding of biological pathways.

The first session was devoted to the current status of the use of experimental information to create biological pathways databases in existing consortia/projects. The second session focused on how activities in biological pathways are implemented

Copyright © 2004 John Wiley & Sons, Ltd.

in existing or developing bioinformatics infrastructures. The third session covered the design, creation and formalization of biological pathways databases. The fourth session was entitled 'Generating and supporting pathway data' and focused on experimental data that support interpretation of biological pathways and/or can be used to generate biological pathways. The fifth session approached the technical aspects of database interoperability and required standards. The last session was dedicated to future plans and perspectives.

The workshop brought together scientists and bioinformaticians who are involved in major multidisciplinary projects as well as in the development of functional genomics databases.

Since some of the participants have contributed reviews or papers to this special section and some extended abstracts are included at the end of this report, we will summarize the main presentation and discussion points here.

### Session 1: Current status of the use of experimental information to create biological pathways databases in existing consortia/projects

The session started with a report on the 2002 ESF workshop on Molecular Networks [7], provided by **Sergio Nasi** (Istituto di Biologia and Patologia Molecolari Consiglio Nazionale delle Ricerche, Università La Sapienza, Roma, Italy). He discussed the molecular complexity that proteins display in living systems. He described some of the theoretical approaches that are being developed to represent and model molecular networks. It was apparent from the discussion that most of the modelled systems are based on known experimental data and that there are therefore no real predictions yet. The need for closer communication between biologists and theoreticians was also mentioned. Sergio Nasi has contributed an overview of available web resources and experimental techniques for studying pathways, and current approaches to modelling the regulatory pathways of the cell [19].

The following speakers reported on the progress made by four EU-funded projects since the Geneva ESF workshop in 2001. These projects are funded under the Fifth Framework Programme (FP5) by the Quality of Life and Management of Living Resources Programme of the European Commission [5]. Descriptions of the projects can be found on the Community R&D Information Service (CORDIS) server: **http://www.cordis.lu/**

**Uwe Kärst** (Gesellschaft für Biotechnologische Forschung, Germany) presented the final status of the REALIS consortium. This project aimed for a postgenomic analysis of the Gram-positive human and animal pathogen *Listeria monocytogenes*. More details on the REALIS project are given in an extended abstract included at the end of this report. He described RibDB, the database created during the project. As is the case for other FP5 EU projects, there is no funding to maintain such databases at the end of the project. The discussion highlighted the need for long-term funding plans for maintaining and updating such databases, which are useful to the entire scientific community.

**Ramon Alonso Allende** (Protein Design Group, Centro Nacional de Biotecnologia, Madrid, Spain) discussed the bioinformatics environment of the REGIA (Regulatory Gene Initiative in *Arabidopsis*) consortium. The consortium members accumulate experimental proteome and genome data, and data from phenotypic analysis of mutant and transgenic species, expression arrays and metabolic analyses. The data are made available through integration into the PlaNet network (see below). The challenges of generating an appropriate database model and design include coping with rapidly evolving experimental technologies and making biologists aware of the constraints of database development.

The progress made by the PlaNet consortium of European plant databases was presented by **Heiko Schoof** (Technische Universität München, Germany) and is reported in a review in this special section [20].

While presenting the work of the BACELL consortium, **Colin Harwood** (Newcastle University, UK) highlighted a number of issues that many databases are facing. One problem is quality control of the data, which are usually not peer-reviewed. Since data are generated using different experimental approaches and technologies, data are sometimes considered as good, and trustworthy, if a correlation exists between various datasources. In many consortia, there is a problem accommodating experimental data generated towards the end of the project, and the time available for data-mining activities is often reduced to a minimum.

Copyright © 2004 John Wiley & Sons, Ltd. 

### Session 2: Pathways as part of bioinformatics infrastructures

**Paolo Romano** (Istituto Nazionale per la Ricerca sul Cancro, Genoa, Italy) discussed the issues involved in creating a network of biological resources such as EBRCN (European Biological Resources Centres Network) (**http://www.ebrcn.org**). Combining persistence of the information with heterogeneity of the data, formal descriptions of links between databases or data sources are necessary. EBRCN has a follow-up in the CABRI (Common Access to Biological Resource and Information [3]) EU project. The CABRI project is described in more detail in a review in this special section [21].

The development of LIMas (Laboratory Information Management for Array Systems: **http://www.mgu.har.mrc.ac.uk/microarray/limas/**) was described by **Sarah Webb** (Oxford University, UK). In developing this system her group has considered standardization for file and information exchange. The development of the system has followed the MIAME compliance recommendation [13], and PEDRO [14] is also considered for the handling of proteomics data. An extended abstract of the presentation is included at the end of this report.

The Data Integration, Analysis and Logistics (DIAL) project of the Center for Medical Systems Biology, in The Netherlands, was described by **Johannes Van Beek**. In a contribution to this special section he describes the DIAL approach in detail [22].

**Anne Morgat** (INRIA Rhône-Alpes, France) discussed ways to represent pathways from different points of view. One common approach is to describe them with components and relationships principles. The issues of interoperability of databases and of methods were illustrated with the Genostar environment (**http://www.genostar.org**) and the GenoExpertBacteria project (**http://www-geb.inrialpes.fr**), which uses the ENZYME and KEGG [11] databases, and the High-quality Automated and Manual Annotation of microbial Proteomes (HAMAP) project (**http://www.expasy.org/sprot/hamap/**).

### Session 3: Design, creation and formalization of biological pathways databases

The environment of the MIPS *Arabidopsis* genome database was discussed by **Heiko Schoof** (Department of Genome-oriented Bioinformatics, Technische Universität, München, Germany). He started with a list of functional requirements that should be apprehended when starting a database integration project. He presented the MIPS *Arabidopsis thaliana* DataBase (MatDB) (**http://www.mips.biochem.mpg.de/proj/thal/db/**), a federated database that makes available, through a common interface, genomic information from various databases. More details on MAtDB can be found in the extended abstract at the end of this report. He introduced MetaMIPS, which integrates public data sources on signal transduction, metabolic pathways and protein–protein interactions and attempts to build metabolic pathways. He also presented the Genome Research Environment (GenRE) project as a flexible workhorse for the annotation of genome information. All genomes being annotated by the MIPS group will move to GenRE (**http://mips.gsf.de/genre/proj/genre/**) to allow for comprehensive annotation of complex genomic features. The discussion that followed covered the quality of data made available. It seems there is a need to apply quality criteria before data can be deposited in public databases.

**Kristian Axelsen** (Swiss Institute of Bioinformatics, Switzerland) described ENZYME (**http://us.expasy.org/enzyme/**), a repository of information on nomenclature of enzymes based on the International Union of Biochemistry and Molecular Biology (IUBMB) recommendations [8]. The CAS registry numbers are also considered for the description of involved chemicals. He pointed out the currently slow process of attributing new classifications. He discussed some open issues, such as the missing link to systematic information on pathways, and the difficulties in controlling accurate propagation of corrections or updates of EC numbers and of general information. As the reactivity of an enzyme is dependent on its biological environment and thermodynamic conditions, the experimental conditions should be present in the description of an enzyme activity. Kristian also introduced the IntEnz project (**http://www.ebi.ac.uk/intenz/index.html**), which aims to act as central repository for enzyme nomenclature that will integrate information from ENZYME, BRENDA and IUBMB.

**Dietmar Schomburg** (Universität Köln, Germany) presented BRENDA (**http://www.brenda.uni-koeln.de/**), a well-established comprehensive

Copyright © 2004 John Wiley & Sons, Ltd. 

Enzyme Information System. It contains information on enzyme reaction and specificity, structure, function, isolation procedure, stability, taxonomic occurrence, etc. The data, which is extracted from original literature, is manually evaluated by scientists, thus reducing the amount of low quality data.

TRANSPATH® (**http://www.biobase.de/pages/products/transpath.html**), a signal transduction pathway database tightly linked to the TRANSFAC® (**http://www.biobase.de/pages/products/transfac.html**) transcription factor database, was introduced by **Claudia Choi** (Biobase GmbH, Germany). Information from TRANSPATH® can be visualized using PathwayBuilder™. In an article in this special section, Claudia describes TRANSPATH® and PathwayBuilder™ in more detail [23].

**Ulrike Wittig** (European Media Laboratory, Germany) discussed the EML project to develop an integrated database system for computational analysis and visualization of biochemical pathways. Towards that goal, a classification system of chemical compounds has been developed to support complex queries in the pathway database. She also presented the first version of BioBrowser, a tool that queries chemical compounds by class and subclass types. For more details, see her contribution to this special section [24].

### Session 4: Generating and supporting pathway data

**Ioannis Xenarios** (Serono Pharmaceutical Research Institute, Switzerland) presented DIP, a protein–protein interaction database (**http://dip.doe-mbi.ucla.edu/**). His presentation included several warnings, such as drawing attention to the relative danger of interpreting protein–protein interactions measured in screening experiments as real interactions *in vivo*. Small changes in experimental conditions might drastically change the presence or absence of measured interactions. Furthermore, the definition of a protein–protein interaction might differ according to the context. A protein described in SwissProt and considered only by its primary structure might not correspond to the real interacting partner in an experiment. He also highlighted the general lack of comments on the reliability of experimentally measured interactions in databases.

The future of Swiss-Prot (**http://www.expasy.org/sprot**) and TrEMBL (**http://www.ebi.ac.uk/trembl/index.html**) becoming part of the UniProt project (**http://www.expasy.org/uniprot/**) was discussed by **Amos Bairoch** (Swiss Institute of Bioinformatics, Switzerland). The database has reached a million entries and should double in size in 2–3 years. This is due to the large amount of bacterial genomes sequenced (about one a week). It is estimated that half of the SwissProt entries are enzymes. Information concerning the activity, structure, membership of a pathway, etc., can be found in various fields of each entry. With respect to pathways, an effort is being made to move from the free-text description format and go for a standardization of the representation, as well as having references pointing to pathway databases.

**Djamel Medjahed** (National Cancer Institute-Frederick, USA) presented VIRTUAL2D, a system to generate virtual 2D maps of proteins, and the Tissue Molecular Anatomy Project (TMAP), a comparative cancer proteomics knowledge base. These tools have been used to develop a collection of tissue-specific plots based on the differential gene expression in the normal and diseased state, gathered from publicly accessible data. His review in this special section has more details on VIRTUAL2D and TMAP [25].

### Session 5: Interoperability of databases with other external databases and standards

**Philipp Reiser** (University of Wales, Aberystwyth, UK) approached gene function by performing reverse engineering of metabolic pathways in yeast. Starting from information in KEGG and IUPAC chemical nomenclature [9], he modelled biochemical reactions and built relations between genes and pathways. This method should be able to browse the relationship between nutrients and biochemically synthesized compounds. He also described the Robot Scientist, a system that helps in the design of experiments and management of a liquid handling system using machine learning.

A method to extract bioentities and relationships from Medline abstracts was presented by **Christian Blaschke** (Protein Design Group, Centro Nacional de Biotecnologia, Madrid, Spain). He pointed out the difficulties of extracting consistent information from text. The nomenclatures for genes are variable and confused, different genes are represented as homonymous abbreviations, different words are

Copyright © 2004 John Wiley & Sons, Ltd.

used in different scientific communities for the same bioentity, and gene names are often represented as nested terms. Ways to look for sets of terms in sentences, instead of only terms, were discussed.

Unfortunately, **Henning Hermjakob** was ill, so **Ioannis Xenarios** gave the talk about the Proteomics Standards Initiative (PSI: **http://psidev.sourceforge.net**). PSI is a bioinformatics initiative of the Human Proteome Organization (HUPO) that develops standard data formats, representation, exchange and annotation in proteomics. In collaboration with the MGED consortium [12] and the American Society for Testing and Materials (ASTM) [1], it proposes recommendations and an XML format for data exchange. It orientates its activities along three complementary axes: protein–protein interactions, mass spectrometry and general proteomics.

**Frederique Lisacek** (GeneBio, Switzerland) presented an innovative approach to shaping biological knowledge. She described environments to improve characterization of protein annotation by combining information from various complementary sources, i.e. databases, prediction tools and expert human input. More details can be found in her contribution to this special section [26].

### Session 6: Perspectives, general discussion

A general discussion took place at the end of the workshop. The topics were chosen according to the discussion points raised in the first five sessions.

### Sustainability of databases

The difficulty of finding funds to maintain and update databases that were financed by EU projects and other time-delimited projects was raised at the Geneva ESF workshop on data integration in 2001 and confirmed again this year. According to comments made in the discussion, UK grants stipulate that databases will be maintained, but it is not explicitly stated where funds for this will come from. On the other hand, it seems that UK employment legislation will affect the career structure of post-docs. Hopefully, this might result in having more scientific officers who could manage such databases. The question of the role of the European Bioinformatics Institute (EBI) was raised. As the EBI has a service role of providing access to databases, it could be involved in the design phase of all databases that might, at the end of a time-defined project, request to be made available on the EBI site. This implies the description and development of guidelines that could include the compatibility conditions for these databases to fulfil. Another approach was to propose that the interested scientific communities, or even societies, should apply and get money to maintain these databases themselves. Maybe an EU Network of Excellence should be created that can tackle the issue of sustainability of biological databases.

### Quality of information in databases, propagation of errors

During the workshop, database developers and providers were frequently asked about the quality of information in their resources. Many databases lack references to the experimental sources of information, which hinders appropriate tracking and interpretation of the biological importance of, and confidence in, the data. Another issue is that the information provided is not always linked to a description of how the data was manipulated, integrated and interpreted between the original experimental source (experiment or original publication) and the final state of the database content. Quality of data and degree of confidence are linked with the consideration that end-users and database providers are familiar with the concept of quoting the limitations and power of specific technologies. It seems that it is not always mandatory for databases to contain biological interpretation of deposited data. However, databases should clearly detail experimental or literature evidences for each piece of data. Proposals for a future workshop that should specifically address the question of data quality in databases were discussed.

### Additional material in the special section

**Javier De Las Rivas** (Centro de Investigación del Cancer, Salamanca, Spain) who was a participant of the workshop but did not give a presentation, has contributed a short review that describes the main experimental and computational approaches to protein–protein interaction networks. He also presents a rationalized scheme of biological definitions that will be useful for a better understanding and interpretation of 'what a protein–protein interaction is'

Copyright © 2004 John Wiley & Sons, Ltd. 

and 'which types of protein–protein interactions are found in a living cell' [27].

### Conclusions

The participants found the workshop interesting, fruitful and in need of a continuation. In general the presentations formed the starting point of many discussions. Questions related to the quality and sustainability of the information provided by consortia or databases arose regularly. It was clear that this theme needs to be addressed in more depth in a future workshop. Technical and functional solutions for facilitating the exchange of information between data sources have to be proposed. The MGED consortium and the PSI initiative present good models of standardization of data and information representation in biological pathway databases. Funding agencies, in particular the EU, should be approached and made aware of the need for the scientific community to have access to funding possibilities for finding solutions to these issues. A decision to submit a proposal for an ESF workshop that addresses the quality issues was approved. It was tentatively decided that Martin Hofmann, Paul van der Vet and Pierre-Alain Binz would be in charge of this proposal.

### Acknowledgements

We would like to thank Mike Taussig and Annette Martin for their support in the organization of the workshop. Thanks also to Jazztime, the Dixieland band that entertained the participants on the Geneva Lake during their trip to the social dinner venue, the restaurant Creux-de-Genthoz. Thanks also to Joan Marsh, who took charge of the notes made during the workshop. Finally, we are deeply thankful to Laure, Claudia, Dolnide and Veronique for the local logistic organization. We are grateful to Uwe Kärst, Heiko Schoof and Sarah Webb who have provided extended abstracts as complementary contributions to this report.

### Extended abstracts

### REALIS

Uwe Kärst
*Gesellschaft für Biotechnologische Forschung, Mascheroder Weg 1, D-38124, Braunschweig, Germany*

The REALIS project aimed at a postgenomic analysis of the Gram-positive human and animal pathogen *Listeria monocytogenes*. The scientific objectives were: (a) to study the evolution of a pathogenic organism by comparative genomics of *L. monocytogenes, L. innocua* and clonally successful pathovariants; (b) the development of postgenomic strategies to provide a complete picture of the remarkable adaptive abilities of this food-borne pathogen; (c) the understanding of the molecular mechanisms by which environmental clues are perceived and translated into adaptive responses; and (d) the establishment of an integrated bioinformatic database incorporating information on biological pathways [10].

A central research area was the identification of regulatory networks in *Listeria*. This work primarily focused on virulence-associated proteins, starting with the known virulence gene cluster and its central transcriptional regulator, PrfA. As *in silico* analyses indicated a significantly larger extent of this regulatory unit, transcriptomics and proteomics analyses were carried out to define the complete PrfA regulon. Comparing several mutants and growth conditions, three groups of genes could be defined that responded, e.g. to the amount of PrfA; there was, however, only a partial overlap in the genes or proteins identified by the two strategies. Furthermore, several genes were obviously only indirectly regulated by PrfA, as no specific promoter could be detected upstream of these genes. A large number of these genes are very likely controlled by the alternative sigma factor B, the general stress response regulator that was shown to interact with the regulation of PrfA itself. Transcriptomics and proteomics analyses of the SigB regulon identified 106 genes controlled by SigB, one of which had no SigB-specific promoter, and only partially overlapping with the data obtained for the PrfA regulon. Therefore, this analysis was extended to include SigmaECF, which is active under microaerophilic growth conditions. However, none of the genes identified in a transcriptome analysis of this regulon were observed as being under the control of PrfA as well. A quite unexpected extension of this list arose from the analysis of *agl*, an *agr*-like locus involved in quorum sensing. The loss of *agl* impairs invasiveness and intracellular proliferation, and again a transcriptome analysis revealed that a number of known virulence factors are part of the Agl

Copyright © 2004 John Wiley & Sons, Ltd. 

regulon. These data indicate that a wide regulatory network exists for the regulation of virulence, obviously with many indirect and currently unknown connections. Sequence and expression data were collected in a central database, RibDB, that contains the data generated by the REALIS consortium.

### MAtDB

Heiko Schoof
*Department of Genome-oriented Bioinformatics, Centre of Life and Food Science, Technische Universität München, D-85350 Freising-Weihenstephan, Germany*

MIPS participated in the first analysis of the completed *Arabidopsis* genome and maintains an online database for that data, MAtDB (**http://mips.gsf.de/proj/thal/db** [15,17,18]). This has been the model dataset and backbone for integration of data from various plant species. In our view, a genome is just a parts list. Once the set of genes is available, their interactions and regulations are what define a plant's lifestyle.

Two major challenges have to be faced in order to efficiently exploit the plethora of data. On the one hand, heterogenous data must be conveniently available in an integrated, comprehensive yet easy-to-access genome knowledge resource. This involves keeping data current, designing data models that can evolve with new data, simple integration of external data, comprehensive views and simple access for humans and applications. On the other hand, analysis methods are required to discover new knowledge from the data.

MAtDB implements automatic update procedures to harvest new data from public databases, while ensuring high data quality by manual curation of inconsistencies. Flexible data modelling based on XML transformations and modular architecture try to address the problem of keeping pace with evolving data. To integrate within a federated system that also allows application-level access, MAtDB implements BioMOBY (**http://www.biomoby.org**)-compliant web services. This is also the basis for building an integrated biological knowledge resource for plant genome data within the European PlaNet project (**http://www.eu-plant-genome.net** [16]).

### Integrating data across platforms

Sarah Webb
*Oxford University Begbroke Science Park, Centre for Ecology and Hydrology, Mansfield Road, Oxford OX1 3SR, UK*

Since the advent of genomic and proteomic technologies, the volume of data being generated by experimentalists has increased exponentially, resulting in a continual need for development of data storage and integration solutions. Genomic data is extremely complex in structure, and much of the data is difficult to represent properly, even in complex relational formats, as there is a lack of recognized data standards and specialist tools to manipulate and view it.

The Environmental Genomics Thematic Programme Data Centre (**http://envgen.nox.ac.uk/**) aims to serve as a local repository for data, formatting it according to international standards and submitting it to the appropriate public databases. To be able to achieve this we need:

- Understanding of, and agreement on, what data and annotations should be provided.
- A standard format of data exchange.
- Development of standard vocabularies and ontologies for describing microarray experiments and samples (also applicable to proteomics data).
- Development of standard protocols, reference samples, controls and data normalization methods.

The mission of this thematic programme is to use existing and emerging genomic and proteomic knowledge and technology to gain a better understanding of ecosystem structure and function. This is being implemented through the funding of projects that address fundamental ecological and evolutionary questions in environmentally important organisms, ranging from microbes to vertebrates. The programme has also invested in a proactive data management initiative, which combines open-source and commercial bioinformatics solutions for analysing, storing, distributing and mining genomic data. Repositories will be implemented that capture data from sources such as LIMaS (a LIMS tool designed specifically for arrays that is MIAME supportive for ESTs, microarrays, proteomics, etc.) and will be tied together by a shared set of meta-data. To facilitate this, we will directly

Copyright © 2004 John Wiley & Sons, Ltd. 

provide software suitable for genomic analysis through the development of Bio-Linux, a version of Linux that is customized for bioinformatics research. Bio-Linux can be downloaded via the Internet (**http://envgen.nox.ac.uk/biolinux.html**).

### References

1. American Society for Testing and Materials: **http://www.astm.org/**
2. Binz P-A, Martin A, Taussig M, de Daruvar A. 2002. Conference Report. The ESF Programme on Integrated Approaches for Functional Genomics workshop on 'Data Integration in Functional Genomics and Proteomics'. *Comp Funct Genom* **3**: 16–21.
3. CABRI: **http://www.cabri.org**
4. European Science Foundation Programme on Integrated Approaches for Functional Genomics: **http://www.functionalgenomics.org.uk**
5. 5th Framework Programme Quality of Life and Management of Living Resources Programme: **http://europa.eu.int/comm/research/quality-of-life.html**
6. Geneva workshop report: **http://www.functionalgenomics.org.uk/sections/activitites/Reports/report_geneva_2001.htm**
7. Granada workshop report: **http://www.functionalgenomics.org.uk/sections/activitites/Reports/report_granada_2002.htm**
8. IUBMB Recommendations: **http://www.chem.qmul.ac.uk/iubmb/**
9. IUPAC chemical nomenclature: **http://www.chem.qmw.ac.uk/iupac/**
10. Kärst U. 2002. REALIS: Postgenomic analysis of *Listeria monocytogenes*. *Comp Funct Genom* **3**: 32–34.
11. KEGG: **http://www.genome.ad.jp/kegg/**
12. MGED consortium: **http://www.mged.org**
13. MIAME: **http://www.mged.org/Workgroups/MIAME/miame.html**
14. PEDRO: **http://pedro.man.ac.uk/home.shtml**
15. Schoof H. 2003. Towards interoperability in genome databases: the MAtDB (MIPS *Arabidopsis thaliana* Database) experience. *Comp Funct Genom* **4**: 255–258.
16. Schoof H, Ernst R, Mayer KFX. 2004. The PlaNet consortium: a network of European plant databases connecting plant genome data in an integrated biological knowledge resource. *Comp Funct Genom* **5**: 000–000.
17. Schoof H, Ernst R, Nazarov V, *et al*. 2004. MIPS *Arabidopsis thaliana* Database (MAtDB): an integrated biological knowledge resource for plant genomics. *Nucleic Acids Res* **32**: D373–376.
18. Schoof H, Zaccaria P, Gundlach H, *et al*. 2002. MIPS *Arabidopsis thaliana* Database (MAtDB): an integrated biological knowledge resource based on the first complete plant genome. *Nucleic Acids Res* **30**: 91–93.
19. Nasi S. 2004. From databases to modelling of functional pathways. *Comp Funct Genom* **5**: 179–183.
20. Schoof H, Ernst R, Mayer KFX. 2004. The PlaNet consortium: a network of European plant databases connecting plant genome data in an integrated biological knowledge resource. *Comp Funct Genom* **5**: 184–189.
21. Romano P, Aresu O, Manniello MA, *et al*. 2004. Interoperability of *CABRI* services and biochemical pathways databases. *Comp Funct Genom* **5**: 169–172.
22. van Beek JHGM. 2004. Data integration and analysis for medical systems biology. *Comp Funct Genom* **5**: 201–204.
23. Choi C, Krull M, Kel A, *et al*. TRANSPATH® — a high quality database focused on signal transduction. *Comp Funct Genom* **5**: 163–168.
24. Wittig U, Weidemann A, Kania R, *et al*. Classification of chemical compounds to support complex queries in a pathway database. *Comp Funct Genom* **5**: 156–162.
25. Medjahed D, Lemkin PF, Smythers GW, *et al*. 2004. Looking for cancer clues in publicly accessible databases. *Comp Funct Genom* **5**: 196–200.
26. Lisacek F, Chichester C, Gonnet P, *et al*. 2004. Shaping biological knowledge: applications in proteomics. *Comp Funct Genom* **5**: 190–195.
27. De Las Rivas J, deLuis A. 2004. Interactome data and databases: different types of protein interaction. *Comp Funct Genom* **5**: 173–178.

Copyright © 2004 John Wiley & Sons, Ltd. 
